# Targeting the Heme Oxygenase 1/Carbon Monoxide Pathway to Resolve Lung Hyper-Inflammation and Restore a Regulated Immune Response in Cystic Fibrosis

**DOI:** 10.3389/fphar.2020.01059

**Published:** 2020-07-14

**Authors:** Caterina Di Pietro, Hasan H. Öz, Thomas S. Murray, Emanuela M. Bruscia

**Affiliations:** Department of Pediatrics, Yale University School of Medicine, New Haven, CT, United States

**Keywords:** monocyte/macrophages, heme oxygenase-1 (HO-1), carbon monoxide (CO), CO-releasing molecules, lung inflammation, cystic fibrosis (CF)

## Abstract

In individuals with cystic fibrosis (CF), lung hyper-inflammation starts early in life and is perpetuated by mucus obstruction and persistent bacterial infections. The continuous tissue damage and scarring caused by non-resolving inflammation leads to bronchiectasis and, ultimately, respiratory failure. Macrophages (MΦs) are key regulators of immune response and host defense. We and others have shown that, in CF, MΦs are hyper-inflammatory and exhibit reduced bactericidal activity. Thus, MΦs contribute to the inability of CF lung tissues to control the inflammatory response or restore tissue homeostasis. The non-resolving hyper-inflammation in CF lungs is attributed to an impairment of several signaling pathways associated with resolution of the inflammatory response, including the heme oxygenase-1/carbon monoxide (HO-1/CO) pathway. HO-1 is an enzyme that degrades heme groups, leading to the production of potent antioxidant, anti-inflammatory, and bactericidal mediators, such as biliverdin, bilirubin, and CO. This pathway is fundamental to re-establishing cellular homeostasis in response to various insults, such as oxidative stress and infection. Monocytes/MΦs rely on abundant induction of the HO-1/CO pathway for a controlled immune response and for potent bactericidal activity. Here, we discuss studies showing that blunted HO-1 activation in CF-affected cells contributes to hyper-inflammation and defective host defense against bacteria. We dissect potential cellular mechanisms that may lead to decreased HO-1 induction in CF cells. We review literature suggesting that induction of HO-1 may be beneficial for the treatment of CF lung disease. Finally, we discuss recent studies highlighting how endogenous HO-1 can be induced by administration of controlled doses of CO to reduce lung hyper-inflammation, oxidative stress, bacterial infection, and dysfunctional ion transport, which are all hallmarks of CF lung disease.

## Introduction 

The hallmarks of cystic fibrosis (CF) lung disease are mucus obstruction, chronic hyper-inflammation, chronic infections, and excessive oxidative stress, which severely damage lung tissue over time and ultimately lead to lung failure. Several anti-inflammatory pathways are compromised in CF (Cantin et al., 2015), which further perpetuates lung inflammation. Despite the role of inflammation in CF lung disease, corticosteroids, or high-dose ibuprofen are the only approved long-term treatments for CF airway inflammation. Both treatments are often poorly tolerated (Mogayzel et al., 2013; Cantin et al., 2015). In addition, when treating CF lung disease, a fine balance must be maintained between dampening the pro-inflammatory response and preserving the host defense against microorganisms (Konstan et al., 2014). This situation calls for novel therapeutic targets, which allow a potent anti-inflammatory/antimicrobial host defense followed by restoration of lung tissue homeostasis.

One of the defective anti-inflammatory pathways in CF is the heme-oxygenase-1/carbon monoxide (HO-1/CO) signaling pathway. The stress response enzyme HO-1 catabolizes heme groups to CO and biliverdin, both strong anti-inflammatory and antioxidant agents. CO also has potent bactericidal properties, and acts in a positive feedback loop to increase HO-1 expression. Due to these combined anti-inflammatory and bactericidal properties, modulation of this pathway is an attractive target in CF.

Here, we will discuss: (1) how the shortcomings of CF lung immunity perpetuate inflammatory signaling and poor bacterial clearance; (2) the role of the HO-1/CO signaling pathway; and (3) the potential of CO-based therapy to reduce lung hyper-inflammation, counteract oxidative stress, and improve bacterial clearance, ultimately restoring lung homeostasis in CF lung disease.

## CF Lung Disease and Inflammation

Lung hyper-inflammation in CF patients starts early in life and is likely driven by accumulation of viscous mucus in the airways (Montgomery et al., 2017). Mucus airway obstruction and impaired mucociliarly clearance create a favorable environment for respiratory infections (Khan et al., 1995; Brennan et al., 2009; Sturges et al., 2010), which intensify the lung inflammatory response. In the early years, infections by *Haemophilus influenza* and *Staphylococcus aureus* (*S. aureus*) are predominant. Over time, the CF airways become susceptible to chronic infections by the opportunistic pathogen *Pseudomonas aeruginosa* (*P. aeruginosa*) (Khan et al., 1995; Brennan et al., 2009; Sturges et al., 2010; Elborn, 2016). In addition to the increased bacterial burden, the epithelial cells and immune cells display altered immune sensing *via* pathogen- or danger-associated molecular patterns (PAMPs or DAMPs), which lead to uncontrolled inflammatory responses. This leads to excessive infiltration of neutrophils, which are impaired in clearing the ongoing infection. Furthermore, CF-affected neutrophils have altered apoptosis (Tirouvanziam et al., 2008) and increased neutrophil extracellular trap formation (Gray et al., 2018), which is accompanied by high levels of neutrophil elastase in the airways of CF patients (Garratt et al., 2015; Kanik et al., 2020). This vicious cycle of persistent infections and uncontrolled pro-inflammatory responses also causes severe oxidative stress through the release of reactive oxygen species (ROS) from neutrophils and CF epithelia, and the irreversible damage of lung tissues (Bonfield et al., 1995; Chmiel and Davis, 2003; Davis, 2006; Cantin et al., 2015; Elborn, 2016; Turnbull et al., 2020). The oxidative stress is worsened by the impaired efflux of chloride, bicarbonate, and other solutes (e.g., glutathione) (Galli et al., 2012).

The failure to efficiently resolve the inflammatory response contributes to the development of chronic hyper-inflammation in CF (Cantin et al., 2015; Roesch et al., 2018; Recchiuti et al., 2019). Indeed, resolution of lung inflammation is an active, tightly coordinated process, whereby counterregulatory mechanisms are induced to clear inflammatory cells from sites of infection or injury in order to restore tissue homeostasis. Successful resolution includes arrest of neutrophil tissue infiltration, apoptosis of neutrophils and their subsequent removal [e.g., *via* efferocytosis (Yurdagul et al., 2017)], dampening of pro-inflammatory signals, clearance of pathogens and cell debris, and initiation of tissue repair processes (Headland and Norling, 2015).

Macrophages (MΦs) play a critical role in maintaining lung tissue homeostasis. During an inflammatory response, they acquire different pro- or anti-inflammatory properties and tissue-reparative phenotypes. Upon recognition of pathogens, they shift toward a pro-inflammatory phenotype, recruiting other immune cells and initiating inflammation. As the inflammation progresses, MΦs not only phagocytose pathogens, but also clear apoptotic cells and cell debris from the lungs. With other signals from surrounding cells or from the pathogen, this efferocytosis transforms MΦs into an anti-inflammatory phenotype, thus limiting inflammation and promoting the resolution/termination of inflammation (Doran et al., 2020). Furthermore, in the later phases of lung injury, MΦs tightly coordinate parenchymal repair processes, which are essential for reestablishing tissue homeostasis. Due to these key roles, it is not surprising that many chronic lung inflammatory diseases such asthma, chronic obstructive pulmonary disease (COPD), CF (Bruscia and Bonfield, 2016a; Bruscia and Bonfield, 2016b), and pulmonary fibrosis (Misharin et al., 2017; Reyfman et al., 2019), are associated with abnormal MΦ behavior.

Monocytes/MΦs from CF patients are dysregulated at many levels. *In vitro* and ex vivo studies from patients with CF and animal models of the disease suggest that both inherited factors (lack of functional CFTR) and acquired factors (CF lung environment) contribute to this dysfunction. As a result, monocytes/MΦs fail to properly handle inflammatory triggers (PAMPs, DAMPs, cytokines, growth factors), struggle to resolve inflammation, and fail to clear dead cells, kill bacteria, and adapt to the environment (reviewed in Bruscia et al. [Bruscia and Bonfield, 2016a; Bruscia and Bonfield, 2016b)].

The exact mechanisms for the exaggerated and dysfunctional inflammatory response observed in CF are not fully understood. However, it appears that the fine balance between the pro- and anti-inflammatory regulatory pathways is disrupted, with heightened pro-inflammatory stimuli and reduced counter-regulatory response, which would ordinarily promote resolution of inflammation. Ibuprofen (Konstan et al., 1995), glucocorticosteroids (Ross et al., 2009), mucolytics (Paul et al., 2004), and antibiotics (Ratjen et al., 2012) are all treatments that have improved CF lung disease and are associated with a reduction in lung inflammation. However, there are concerns about using anti-inflammatory therapies in chronically infected CF patients. Indeed, blocking induction of inflammation may have immunosuppressive effects that compromise the host defense and thus worsen lung infections. This was observed in clinical studies assessing the effect of the LTB4-receptor antagonist BIIL 284, an inhibitor of neutrophil migration, in children and adults with CF. This study was terminated prematurely due to a significant increase in the frequency of pulmonary exacerbations (due to bacterial infections) in adult patients who received the treatment (Doring et al., 2014; Konstan et al., 2014).

The use of CFTR modulators, which correct mutant CFTR trafficking to the plasma membrane (correctors) and enhance its activity (potentiators), are now FDA-approved for most CF patients (Middleton et al., 2019). However, there are few long-term studies of their impact on immune response and monocyte/MΦs function. While Rowe et al. (2014) reported that the CFTR modulator VX-770 (Ivacaftor) did not reverse lung inflammation, other studies showed partial reduction of lung inflammation (Hisert et al., 2017). Ex vivo studies on monocytes and monocyte-derived MΦs from patients with CF suggest that Ivacaftor modulates the inflammatory response (Hisert et al., 2017; Zhang et al., 2018; Jarosz-Griffiths et al., 2020) and improves bacterial killing (Hisert et al., 2017; Riquelme et al., 2017; Barnaby et al., 2018). The newly approved triple combination CFTR modulator therapy elexacaftor/tezacaftor/ivacaftor (Trikafta) (Middleton et al., 2019) has shown great promise for many CF patients. However, its effect on the abnormal inflammatory response in CF has not been fully elucidated, and it is not known whether it will help control lung hyper-inflammation over the longer life expectancy achieved. Moreover, these therapies are not applicable for all mutations and, therefore, for all patients with CF. Thus, novel therapeutic approaches are needed that, in combination with CFTR modulators, will rescue the abnormal anti-inflammatory regulatory mechanisms and facilitate the resolution of the inflammatory response, while maintaining a potent antimicrobial host defense. Below, we discuss the HO-1/CO pathway, which facilitates anti-inflammatory and antioxidant activities while strengthening the host’s bactericidal functions. This pathway is thus an attractive therapeutic target for CF.

## HO-1 Function and Regulation

Heme oxygenases (HO) are enzymes that facilitate the degradation of heme, a ubiquitous molecular complex consisting of iron and tetrapyrrole protoporphyrin IX. The heme from the hemoglobin in red blood cells and myoglobin in muscles is involved in the transport and storage of oxygen, respectively. However, many other proteins also use a heme group for fundamental cellular processes. If released from proteins, an excess of free heme is highly toxic because it promotes oxidative stress (Biswas et al., 2014; Wegiel et al., 2015). HO enzymes thus play a crucial role in cells (Gozzelino et al., 2010). HO activity is represented by two separate isoforms: an inducible isoform HO-1 and a constitutively expressed isoform HO-2. A suspected third isozyme, HO-3, turned out to be a pseudogene derived from processed HO-2 transcripts (Mccoubrey et al., 1997). HO-1 and HO-2 are the products of distinct genes, *hmox1* and *hmox2*, respectively. They differ in primary amino acid sequence, biochemical and biophysical properties (Cruse and Maines, 1988; Ryter and Choi, 2016). HO-1 is an integral membrane component of the smooth endoplasmic reticulum (Gottlieb et al., 2012), but it is also localized in plasma membrane caveolae (Kim et al., 2004), mitochondria (Converso et al., 2006), and nuclei (Biswas et al., 2014). HO-1 is undetectable under physiological conditions but is highly induced after exposure to a broad range of chemical and physical stimuli including heme, ultraviolet-A radiation, hydrogen peroxide, redox cycling compounds, heavy metals, hypoxia, hyperoxia, cytokines, hormones, growth factors, and microorganisms. HO‐1 is mainly induced in hepatic, endothelial, myeloid, and respiratory epithelial cells. One exception is the spleen, where constantly high levels of HO-1 ensure an efficient recycling of iron from senescent erythrocytes (Ryter et al., 2006). Monocytes/MΦs rely on abundant induction of the HO-1 for a controlled immune response and for potent bactericidal activity. In liver endothelial and epithelial cells HO-1 plays a critical anti-oxidant and pro-survival function in response to cellular stressors (Ryter et al., 2006). In contrast to HO-1, HO-2 is constitutively expressed in most tissues, including brain, testis, endothelial, and smooth muscle cells (Zakhary et al., 1996), and is refractory to HO-1 inducers (Maines et al., 1986). The inducible nature of HO-1 makes it an attractive target for drug discovery.

**Figure 1:**
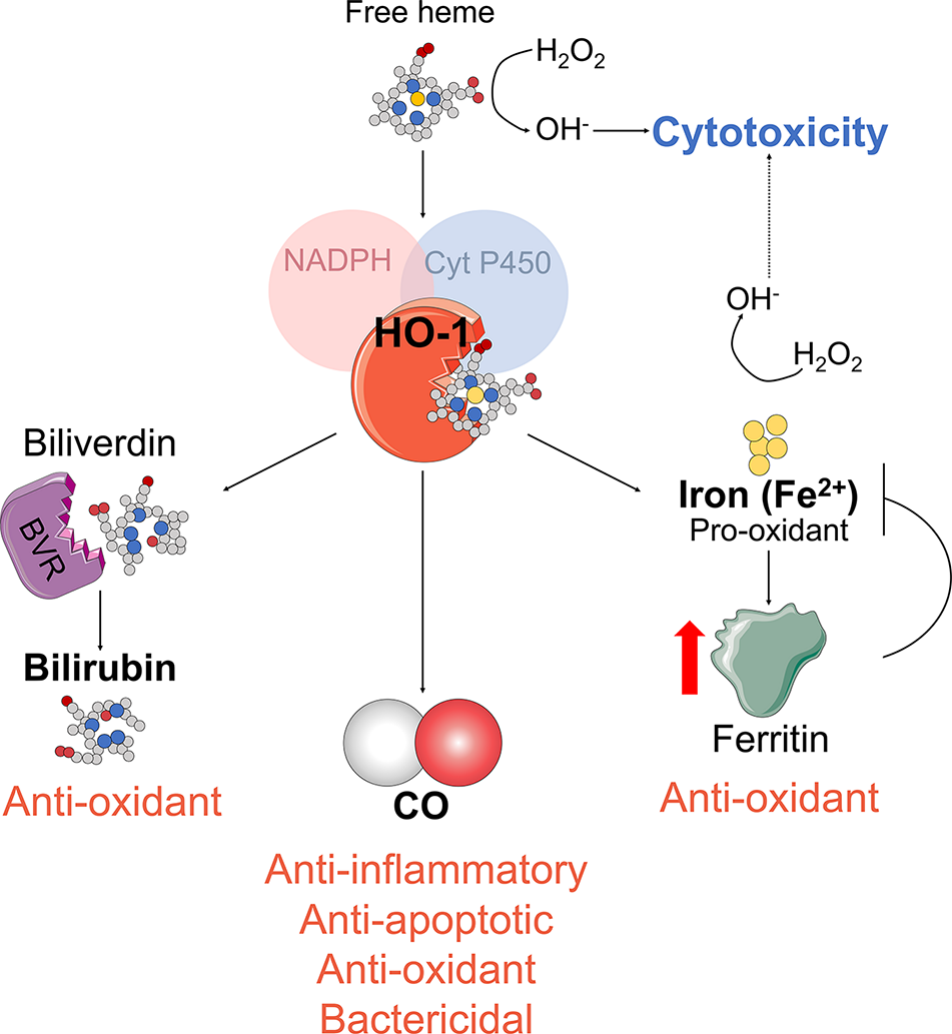
HO-1 enzymatic activity. HO-1 enzymatic activity generates biliverdin and releases carbon monoxide (CO) and Fe^2+^. Biliverdin is transformed into bilirubin by the biliverdin reductase (BVR). Fe^2+^ is sequestered by the iron storage protein ferritin.

HO-1 catalyzes the first and rate-limiting step in the oxidative catabolism of heme groups. With the use of cytochrome P-450, nicotinamide adenine dinucleotide phosphate (NADPH), and molecular oxygen, HO-1 catabolizes heme into equimolar amounts of carbon monoxide (CO), free iron (Fe^2+^), and biliverdin IXa. The cytoprotective effects of HO-1 are enhanced by its catabolites. Biliverdin is rapidly metabolized to bilirubin (a highly antioxidant compound) by the biliverdin reductase. The free iron, which can stimulate free radical formation, is promptly sequestered by ferritin and recycled for heme synthesis. Degradation of heme is the only mammalian pathway known to produce CO. This gaseous molecule is toxic at higher concentrations because it binds hemoglobin and thus prevents the transport of oxygen. However, at physiological concentrations, CO has strong cytoprotective, anti-inflammatory, antioxidant, and bactericidal properties (**Figure 1**) (Motterlini and Otterbein, 2010; Ryter et al., 2018).

The essential cytoprotective role of HO-1 has been demonstrated by the phenotype of HO-1-null mice (HO-1 KO), which display increased embryonic lethality, anemia, and chronic inflammatory disorders. Cells isolated from HO-1 KO animals are also more susceptible to oxidative stress (Poss and Tonegawa, 1997a; Poss and Tonegawa, 1997b). HO-1 KO animals display increased mortality after lipopolysaccharide (LPS) administration, which supports the importance of HO-1 in mediating protection during bacterial infection (Wiesel et al., 2000). Importantly, the phenotypical alterations in the uniquely observed case of human HO-1 deficiency are similar to those in HO-1 KO mice, with severe hemolytic anemia, endothelial degradation, reduced serum bilirubin, renal and hepatic iron accumulation, and a pro-inflammatory phenotype (Yachie et al., 1999).

The expression of HO-1 is regulated primarily at the transcriptional level *via* regulatory element sites localized at the 5′-untranslated region of the *hmox1* gene promoter. These include binding sites for several prominent transcriptional factors (TFs), such as nuclear factor E2–related factor-2 (Nrf2) (Alam et al., 1999), activator protein-1 (AP-1) family (Alam and Den, 1992; Alam et al., 1999), nuclear factor-kappa B (NF-*k*B) (Lavrovsky et al., 1994), and hypoxia-inducible factor-1 alpha (HIF-1α) (Lee et al., 1997). For a comprehensive review of HO-1 regulation, please refer to (Alam and Cook, 2007; Waza et al., 2018; Medina et al., 2020). Many of the TFs and signaling pathways involved in modulating HO-1 expression are dysregulated in CF cells, resulting in decreased HO-1 induction (discussed in *HO-1 Dysregulation in CF*).

**Figure 2:**
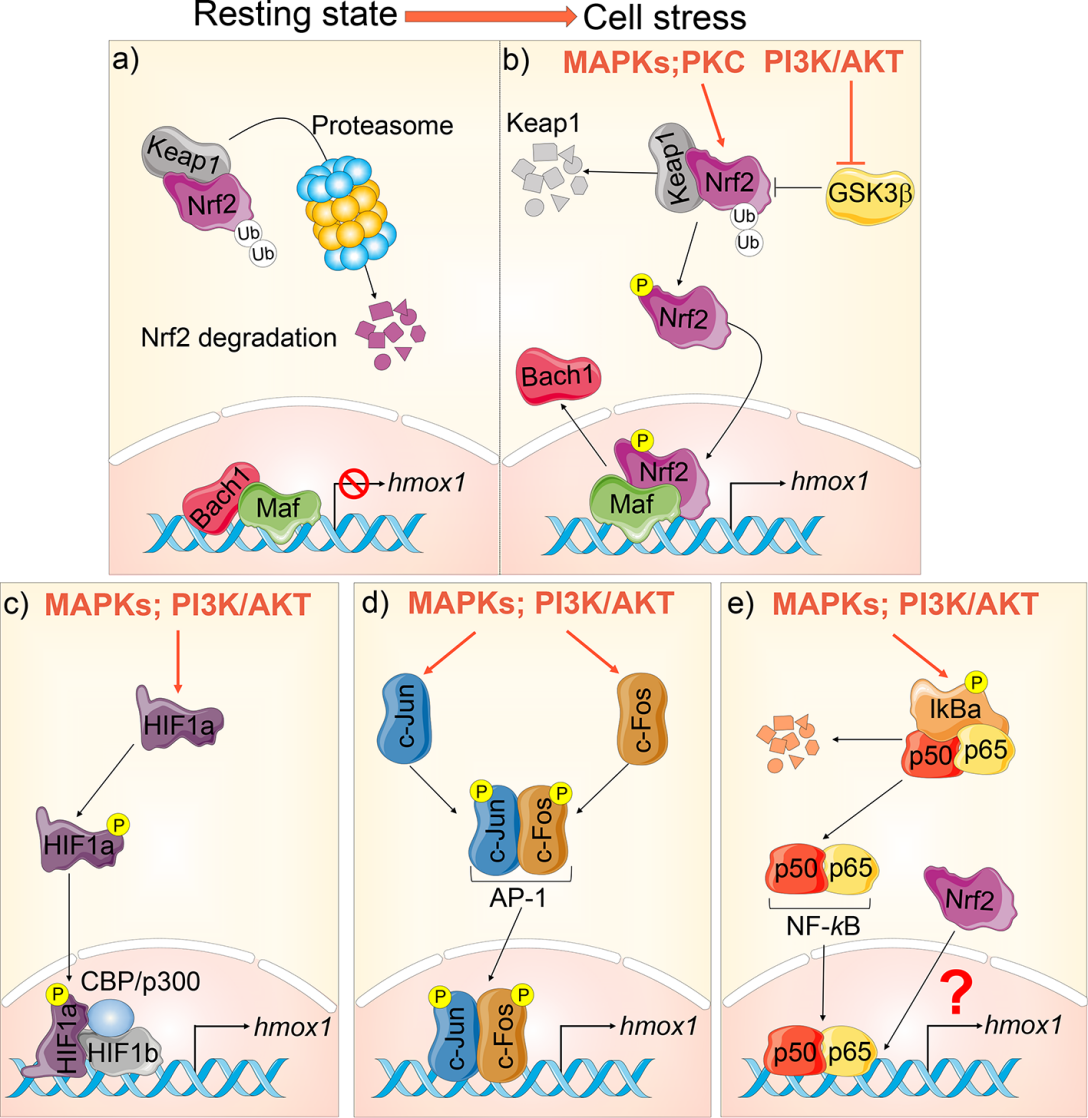
*Hmox1* transcriptional regulation. **(A)** At steady state, Nrf2 is bound to Keap1 in the cytoplasm and targeted for proteasomal degradation. Bach‐1 is bound to Maf at the promoter region of the *hmox1*, suppressing its transcription. **(B)** During cellular stress, *hmox1* expression is activated in several ways: mitogen-activated protein kinase (MAPKs) and protein kinase C (PKC) phosphorylate Nrf2. This stabilizes Nrf2, leading to its translocation into the nucleus. PI3K/AKT inhibits GSK3β. When activated, GSK3β facilitates the ubiquitination and proteasomal degradation of Nrf2. Once in the nucleus, Nrf2 displaces Bach‐1 at the *hmox1* promoter leading to transcription. **(C)** HIF-1 is a heterodimer composed of HIF-1α and HIF-1β. HIF-1α phosphorylation leads to its translocation to the nucleus and association to HIF-1β and CBP/p300, thus inducing *hmox1* transcription. **(D)** Phosphorylation of *c-Fos* and *c-Jun* leads to their translocation to the nucleus and formation of the AP-1 complex, which induces *hmox1* expression; **(E)** NF-*κ*B is sequestered in the cytosol under basal conditions by the inhibitor I*κ*B. Phosphorylation results in the proteasomal degradation of I*ĸ*B and the consequent release and nuclear translocation of NF-*κ*B dimers (p50/p65) which targets *hmox1* activation. A complex crosstalk between NF-*κ*B and Nrf2 can also inhibit *hmox1* transcription.

Nrf2 is a major transcriptional regulator of HO-1 (Chan and Kan, 1999). At steady state, Nrf2 localizes in the cytoplasm of the cells, where it is inactivated when associated with its cytosolic repressor Kelch-like ECH-associated protein-1 (Keap1). Keap1 actively promotes Nrf2’s rapid degradation by the ubiquitin–proteasome pathway to ensure low Nrf2 levels in the cell. Exposure to electrophilic or oxidative stresses causes a conformational change in Keap1, with the subsequent dissociation of Nrf2. Nrf2 then translocates into the nucleus, where it forms a heterodimer with small masculoaponeurotic fibrosarcoma (Maf) proteins and binds to the antioxidant response elements (ARE) in the promoter region of genes coding for antioxidant and detoxifying enzymes. These include HO-1, NAD(P)H quinone oxidoreductase 1 (NQO1*)*, glutamate-cysteine ligase (GCL), and glutathione S transferases (GSTs), which all execute antioxidative functions in cells (Bryan et al., 2013). The Nrf2-mediated HO-1 expression is also finely regulated by TF BTB and CNC Homology 1 (Bach1), which also forms heterodimers with Maf and competes with Nrf2 for the binding sites in the *hmox1* promoter region, thus suppressing HO-1 expression (Dhakshinamoorthy et al., 2005). Thus, HO-1 induction is highly regulated and requires the release of Nrf2 from Keap1, the inactivation of its competitor Bach1, and the availability of Maf to initiate transcriptional signaling (Ogawa et al., 2001; Bryan et al., 2013) (**Figures 2A, B**).

Functional binding sites for AP-1 (Alam et al., 1999), HIF-1α (Lee et al., 1997), and NF-*k*B (Lee et al., 1997) have been identified within the promoter region of *hmox1* gene (details in **Figures 2C–E**). In response to hypoxia, HIF-1α is phosphorylated by the MAPK p38. This leads to its translocation to the nucleus, where it associates with HIF-1β, the transcriptional co-activator CREB-binding protein (CBP), and p300, thereby leading to transcription of HO-1 (Lee et al., 1997; Medina et al., 2020). Several studies have shown that NF-*k*B not only positively modulates HO-1 expression by directly binding to its promoter (Naidu et al., 2008; Rushworth et al., 2012), but it may also work as a negative regulator for HO-1 expression. Some of these conflicting data can be reconciled by the complex NF-*κ*B crosstalk with Nrf2 in modulating HO-1 induction during specific cellular responses (Liu et al., 2008; Yu et al., 2011). For instance, NF-*k*B decreases the availability of CBP/p300 for Nrf2, thus preventing Nrf2 transcriptional activity (Liu et al., 2008). This is relevant for CF, where shifting the competitive binding of CBP/p300 in favor of Nrf2 (over NF-*k*B) leads to increased expression of antioxidant and anti-inflammatory genes and decreased cellular inflammation (Ziady et al., 2012). The molecular mechanisms underpinning the dynamic crosstalk between NF-*κ*B and the Nrf2 are extensively reviewed in (Wardyn et al., 2015) and are still under investigation.

Post-transcriptional and post-translational modifications are also potential regulatory mechanisms for controlling HO-1 levels. A number of microRNAs (e.g., miR-24, miR-200c, miR-204, miR-211, miR-155, miR-378, miR-377, miR-217) directly regulate HO-1 levels by decreasing *hmox1* messenger RNA stability or translation. Other miRNAs indirectly modulate HO-1 by affecting the expression of upstream regulatory factors, such as Nrf2, Keap1, and Bach1 [Review in (Cheng et al., 2013)]. The GT-microsatellite polymorphism, located in the proximal human *hmox1* promoter region, also contributes to the regulation of HO-1 expression. These short GT repetitions increase HO-1 expression, which correlates with a reduced risk of developing rheumatoid arthritis, chronic pulmonary emphysema, and other diseases (Exner et al., 2004).

Activation of several signaling cascades mediates induction of HO-1, including the mitogen-activated protein kinase (MAPKs) superfamily (p38, ERK, and JNK), protein kinase C (PKC), and phosphatidylinositol 3-kinase (PI3K) (**Figures 2B–E**). Protein kinase C facilitates Nrf2 nuclear translocation by phosphorylation of Nrf2 at the Keap1 binding site freeing Nrf2 from Keap1 (Huang et al., 2002). MAPKs and PI3K/AKT can directly (*via* phosphorylation) or indirectly regulate transcription factors required for the HO-1 induction. The MAPKs and PI3K/AKT signaling cascades have been extensively investigated in the context of Nrf-2-dependent HO-1 activation. PI3K/AKT signaling can indirectly promote HO-1 transcription by inhibiting glycogen synthase kinase 3β (GSK3β)-mediated phosphorylation and subsequent ubiquitination and proteasomal degradation of Nrf2 (Bryan et al., 2013). Furthermore, PI3K/AKT signaling activation in response to oxidative stress results in actin polymerization and depolymerization, which promotes translocation of actin-bound Nrf2 into the nucleus (Kang et al., 2002).

## HO-1 Dysregulation in CF

In CF, *hmox1* has been reported to be a modifier gene, as a specific *hmox1* allele correlated with improved lung function in pediatric CF patients chronically infected with *P. aeruginosa* (Park et al., 2011). On top of genetic variants, studies comparing nasal epithelial cells and blood cells of CF patients with healthy donors have revealed altered epigenetic modifications of the *hmox1* gene (Magalhaes et al., 2017). An early study showed that the lungs of patients with CF have increased HO-1 expression compared to control lung resections from patients with cancer (Zhou et al., 2004). This is expected given the inflammatory environment of CF lungs. A better control would be tissues from patients with other lung inflammatory conditions. In the same study, the authors provided the first evidence for the beneficial effect of HO-1 in CF cells. Namely, overexpression of HO-1 in CF human bronchial epithelial (HBE) cell lines (IB3.1) led to potent cytoprotective properties against *P. aeruginosa* infections (Zhou et al., 2004). HO-1 expression in CF HBE cell lines (CFBE41o-) was decreased at baseline and its induction was hampered following stimulation by LPS or hypoxia compared with a HBE cell line control (Chillappagari et al., 2014; Chillappagari et al., 2020). This suggests that, while HO-1 can still be induced in the absence of CFTR, the amount produced may not be sufficient to provide beneficial effects. The lack of HO-1 correlates with an increased iron load (Chillappagari et al., 2014), that is also observed in lavages and lung tissues of CF patients (Stites et al., 1999; Ghio et al., 2013) and favors *P. aeruginosa* infections (Reid et al., 2002; Moreau-Marquis et al., 2008).

**Figure 3:**
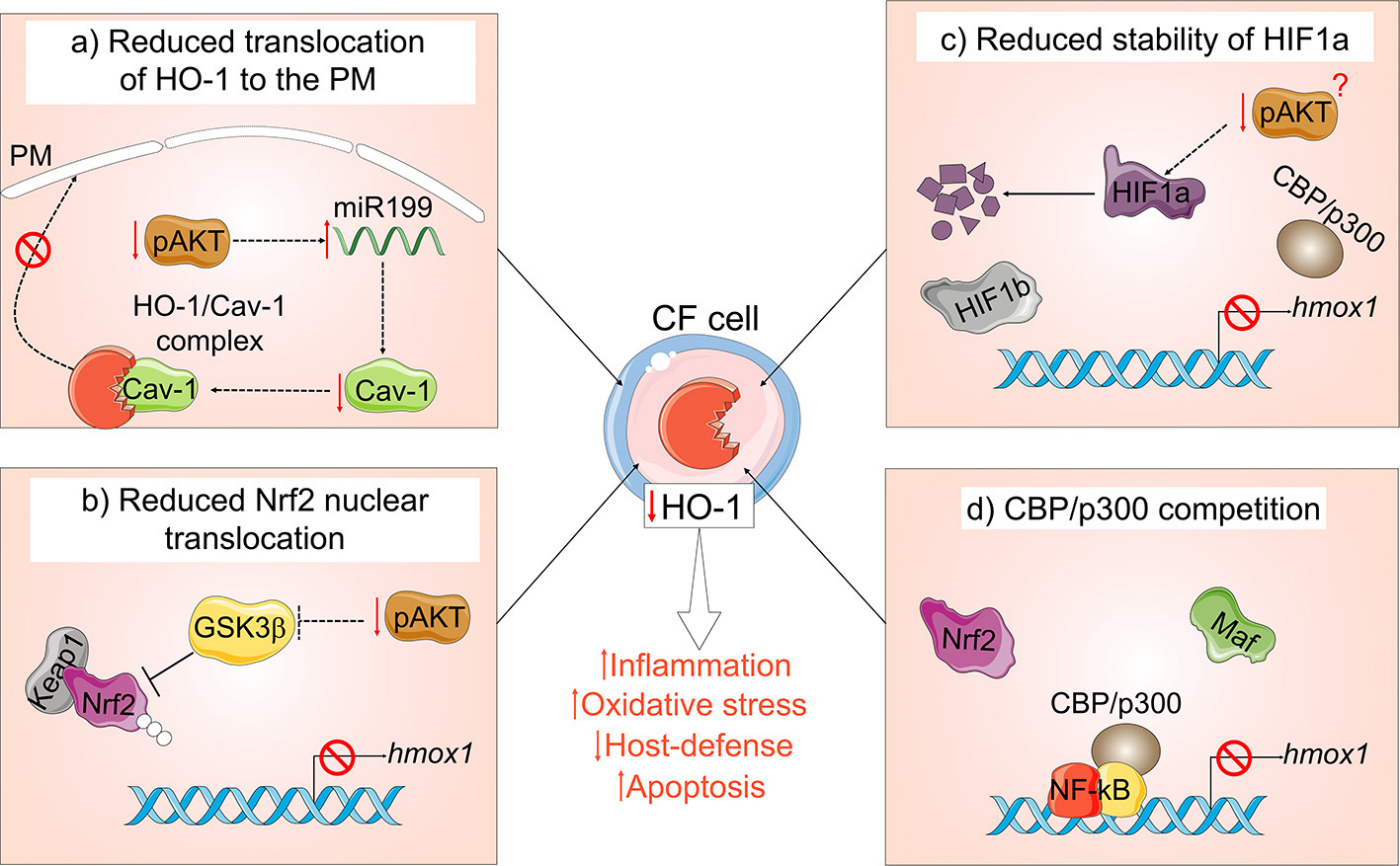
Mechanisms of HO-1 dysregulation in cystic fibrosis (CF). In the absence of functional CFTR: **(A)** MΦs have a blunted PI3K/AKT signaling in response to TLR4 activation, which leads to accumulation of miR-199a-5p, which reduces Cav1 expression. Loss of Cav1 impairs translocation and compartmentalization of HO-1 at the plasma membrane (PM); **(B)** blunted PI3K/AKT signaling in CF cells results in elevated levels of active GSK3β, which leads to Nrf2 ubiquitination and proteasomal degradation and **(C)** affects the stability of HIF-1α. **(D)** NF-*k*B in CF cells competes for the Nrf2 co-activator CBP/p300, thus preventing Nrf2 transcriptional activity.

Several dysfunctional mechanisms may account for the blunted HO-1 induction in CF cells (**Figure 3**). Our group has demonstrated that HO-1 is inefficiently induced in human and murine CF MΦs in response to inflammatory or infectious triggers, which correlate with exaggerated inflammation and prolonged inflammatory signaling (Zhang et al., 2013; Zhang et al., 2015; Di Pietro et al., 2017). We have also shown that the defective induction of HO-1 is due to blunted activation of the PI3K/AKT pathway downstream of toll-like receptor 4 (TLR4) activation in MΦs from CF mouse models and patients with CF. Alteration of this pathway decreases HO-1 expression and perpetuates the inflammatory response. In addition to decreased induction, the HO-1 cellular distribution is altered in CF-affected MΦs, thus diminishing its beneficial effect. In response to LPS, HO-1 normally translocate to plasma membrane lipid rafts in a caveolin 1 (Cav1)- dependent manner, where it destabilizes the binding between TLR4 and its adapter protein myeloid differentiation factor 88 (MyD88) *via* CO production, thus ending inflammatory signaling (Wang X. et al., 2009). We found that HO-1 does not compartmentalize to the cell surface in CF MΦs, but rather accumulates intracellularly due to decreased Cav1 expression (Zhang et al., 2013). We linked the decreased levels of Cav1 expression to high levels of miR-199a-5p (which targets caveolin 1 3’-UTR) downstream of blunted PI3K/AKT signaling in CF MΦs (Zhang et al., 2015). Importantly, modulation of this pathway *via* overexpression of HO-1 or treatment with CO-releasing molecules (discussed in the next section) was sufficient to improve the signaling cascade, thus reducing hyper-inflammation in CF MΦs (Zhang et al., 2013). In investigating how loss of CFTR leads to blunted PI3K/AKT signaling, we found that ezrin, an F-actin binding protein that forms a macromolecular complex with CFTR at the plasma membrane (Guggino and Stanton, 2006), links CFTR, TLR4, PI3K/AKT signaling, and HO-1 expression in MΦs (Di Pietro et al., 2017).

Ziady et al. (Chen et al., 2008; Ziady et al., 2012) showed that CFTR deficiency in HBE cells reduces translocation of the transcription factor Nrf2 into the nuclear compartment, thus impairing the transcription of antioxidant genes, including HO-1. This group also found that the co-activator CBP favors the binding to NF-*k*B (over Nrf2), which increases inflammatory signaling (Chen et al., 2008). Kelley et al. showed that Rp-cAMPS, a cAMP competitor, rescued Nrf2 activity in CF epithelial cells by shifting the CBP association in favor of Nrf2 (over NF-*k*B), thus decreasing inflammatory signaling and increasing antioxidant and anti-inflammatory activity (Ziady et al., 2012). In this study HO-1 expression was not tested, however, the rescued Nrf2 activity should result in increased HO-1 expression. Importantly, Ziady’s group recently showed that the broadly used CFTR modulators VX-809 (Lumacaftor) and VX-661 (Tezacaftor) significantly increase Nrf2 activity after correction of CFTR expression in primary epithelial cells of CF patients with homozygous F508del mutations (Borcherding et al., 2019). VX-809 also stabilizes PTEN association with the mutant CFTR protein (upstream regulator of PI3K) (Riquelme et al., 2017) and increases cellular levels and phosphorylation of ezrin (Matos et al., 2019). This helps the mutant CFTR to form a stable macromolecular complex at the plasma membrane, thus improving function (Abbattiscianni et al., 2016). Plasma membrane stabilized mutant CFTR, restores the CFTR downstream signaling transduction events that reestablish the Nrf2-HO-1 axis in VX809-treated cells (Borcherding et al., 2019). In addition to Nrf2, stabilization of HIF-1α is downregulated in unstimulated and hypoxia-stimulated CFTR-deficient HBE cells, which decreases HO-1 expression (Legendre et al., 2011).

While CFTR modulators decreased the inflammatory response (Hisert et al., 2017; Zhang et al., 2018; Jarosz-Griffiths et al., 2020), and improved their bacterial clearance (Barnaby et al., 2018; Zhang et al., 2018) in monocyte/MΦs from patients with CF, these studies did not investigate a possible increase (and thus beneficial effect) of HO-1 levels. In CF patients, CFTR modulators initially decreased the bacterial burden in the lungs, but the bacteria re-emerged over time (Hisert et al., 2017). Thus, to preserve lung function in patients with CF in the long term, we propose that targeting the HO-1/CO pathway can complement existing treatments.

Several trials and studies have indirectly tested HO-1 induction in patients with CF. The first tested candidate was sulphoraphane, an antioxidant compound that induces Nrf2 signaling and improves bacterial clearance by alveolar MΦs (Harvey et al., 2011). The study found no adverse effects of increased dietary sulphoraphane intake. However, larger studies are needed to test sulphoraphane’s efficacy in CF (NCT01315665). GSK Pharmaceuticals have developed a promising small molecule for therapeutically inducing HO-1 (Davies et al., 2016). This molecule activates Nrf2 by binding the Keap1-Nrf2 binding site, favoring their dissociation. It thus improves opsonic phagocytosis in MΦs isolated from COPD patients (Bewley et al., 2018), and may be beneficial for CF patients. As an alternative approach, exogenous delivery of low doses of CO, a potent inducer of HO-1, can be considered to improve CF lung disease. We have proven that exogenous CO delivery can overcome the defective plasma membrane localization of HO-1 in CF MΦs challenged with LPS (Zhang et al., 2013). However, certain epigenetic changes present at the *hmox1* gene in CF- affected cells (Magalhaes et al., 2017), may reduce the efficacy of such a therapeutic approach. In the following sections, we discuss the cellular effects of CO administration, and its relevance to CF.

## CO Anti-Inflammatory and Antioxidant Functions, and Relevance to CF

Similarly, to nitric oxide (NO) and hydrogen sulfide (H_2_S), CO is a potent gaseous signaling molecule that can freely diffuse through membranes (Fagone et al., 2018). The biological activity of CO depends on its ability to bind with ferrous (Fe^2+^) ions, thus controlling the activity of several heme-containing proteins (e.g., nitric oxide synthase (NOS), NADPH oxidase, cytochrome C oxidase, guanylate cyclase) (Wang et al., 2014; Ji et al., 2016; Motterlini and Foresti, 2017; Ryter et al., 2018). These proteins activate signaling pathways that are implicated in cell protection/survival against stress, in antioxidant responses, and in regulating inflammation. CO’s downstream targets include p38, HIF-1α, PPARγ, glutathione, nitric oxide, and PI3K/AKT, many of which are altered in CF (Cantin et al., 2015). The current understanding of CO’s biological function derives from studies in which cells and animal models were exposed to non-toxic doses of free CO, or were treated with small molecules able to release controlled amounts of CO, i.e., CO-releasing molecules (CO-RMs). CO-RMs were initially engineered by Motterlini and colleagues (Motterlini et al., 2002). They contain a transition metal (e.g., ruthenium, cobalt, iron) surrounded by carbonyl (CO) groups. Different CO-RMs have been developed to minimize toxicity, improve solubility, and increase control of CO release. Exogenous delivery of low doses of CO mimics the physiological/non-toxic effects elicited by the production of endogenous cellular CO (Motterlini and Otterbein, 2010; Wegiel et al., 2013; Wang et al., 2014; Ji et al., 2016; Motterlini and Foresti, 2017; Ryter et al., 2018).

Several *in vivo* studies, including ours in CF-affected mice (Zhang et al., 2013), support the notion that exogenous delivery of CO prevents hyper-inflammation and tissue damage in the context of sepsis, sterile inflammation, and hyperoxia (Motterlini and Otterbein, 2010). CO reduces the number of neutrophils in septic lungs by controlling transendothelial migration (Mizuguchi et al., 2009). Moreover, at low concentrations, CO attenuates the lung inflammatory response in mice challenged with LPS or live bacteria (Macgarvey et al., 2012; Wegiel et al., 2014b; Wilson et al., 2017; Kim et al., 2018). It differentially and selectively inhibits LPS-induced expression of pro-inflammatory cytokines (e.g., TNF-α, IL-6), while increasing levels of anti-inflammatory molecules (e.g., IL-10, IL-1 receptor antagonist (IL-1Ra, PPAR**-**γ) (Bilban et al., 2006; Haschemi et al., 2011; Piantadosi et al., 2011; Macgarvey et al., 2012; Uddin et al., 2015). This is relevant to CF because CF airway epithelial cells (Perez et al., 2007) and CF MΦs (Bruscia et al., 2009) both secrete more pro-inflammatory cytokines during inflammatory stimuli compared to non-CF cells. Moreover, CO can help reestablish the secretion levels of IL-10, which are lower in CF lungs (Bonfield et al., 1995), and of PPAR**-**γ, which are lower in CF epithelial cells and MΦs (Andersson et al., 2008; Harmon et al., 2010). Murine (Sawle et al., 2005) and human (Chhikara et al., 2009) MΦs are particularly responsive to CO treatment, which attenuates the inflammatory response to LPS (Sawle et al., 2005).

The anti-inflammatory effect of CO in response to LPS may be mediated by augmenting the caveolin 1(Cav-1)/TLR4 interaction at plasma membrane caveolae by a p38 MAPK-dependent mechanism (Otterbein et al., 2000; Sawle et al., 2005; Bilban et al., 2006; Chhikara et al., 2009; Tsoyi et al., 2009; Wang X. et al., 2009), which favors termination of pro-inflammatory signal transduction events. Importantly, CF MΦs have increased levels of plasma membrane TLR4 receptors (Bruscia et al., 2011) and decreased Cav1 expression in response to LPS (Zhang et al., 2013). We have shown that low levels of Cav1 prevent translocation of HO-1 to the plasma membrane of activated CF MΦs (Zhang et al., 2013), where it normally localizes (Wang X. et al., 2009). CO-RM treatment reversed this dysfunction in CF cells (Zhang et al., 2013).

**Figure 4:**
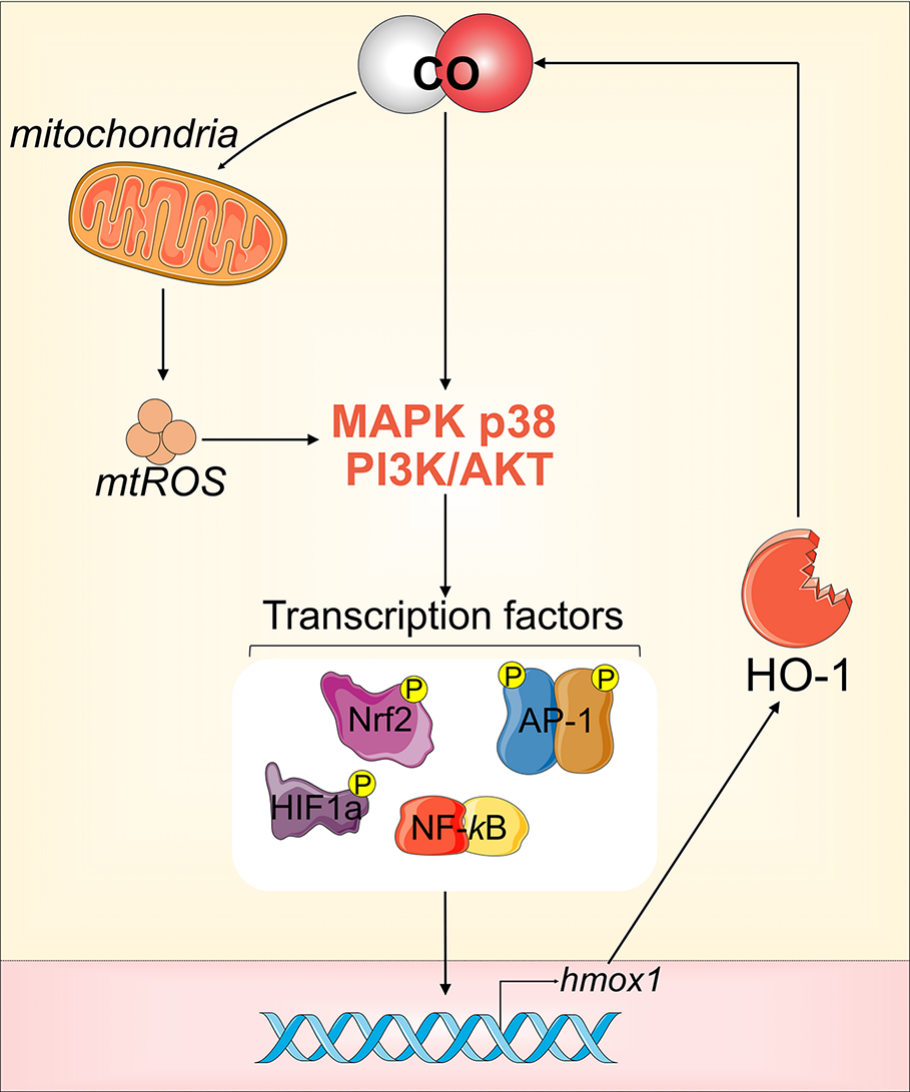
CO-mediated induction of HO-1. Low dose CO can activate all the transcriptional factors (TFs) that drive the expression of HO-1, *via* either direct or indirect activation of MKK3/p38 MAPKs and PI3K/AKT signaling.

CO’s anti-inflammatory effects also rely on its ability to strongly induce expression of endogenous HO-1, thus increasing autonomous, cellular CO production. HO-1 expression is driven by several TFs and upstream signaling events (see *HO-1 Function and Regulation*, **Figure 2**). Exogenous CO administration can activate all the shown/mentioned TFs to induce HO-1 expression, with activation of preferential pathways depending on the experimental conditions, cell type, and cellular stressors (Piantadosi et al., 2008; Zhang et al., 2013) (**Figure 4**). Mechanistically, low doses of CO transiently increase mitochondrial ROS (mtROS) levels (Wang et al., 2007). This temporary increase in mtROS activates the MKK3/p38 MAPKs and PI3K/AKT signaling pathway, which ultimately strongly induces HO-1 expression (Otterbein et al., 2000; Otterbein et al., 2003).

More recently, it has been proposed that CO promotes resolution of inflammation by inducing expression of specialized pro-resolving lipid mediators (SPMs) derived from the metabolism of polyunsaturated fatty acids (Serhan et al., 2014). In a mouse model of peritonitis (Chiang et al., 2013) and a primate model of pneumonia (Dalli et al., 2015), CO induces expression of a key biosynthetic enzyme (12/15-LOX in mice, and 15-LOX-1 in humans), which induces the production of SPMs. In these models, CO increases levels of Resolvin (Rv)-D1, RvD2, and Lipoxin A4 (LXA4), and reduces levels of pro-inflammatory lipid mediators, such as thromboxane B2 (TXB2), leukotriene B4 (LTB4), and prostaglandin E2 (PGE2). This is relevant because CF patients have a reduced capacity to biosynthesize SPMs. Clinical studies have shown that the arachidonic-acid-derived LXA4 is reduced in the bronchoalveolar lavage fluid (BALF) of patients with CF (Karp et al., 2004; Chiron et al., 2008; Yang et al., 2012; Ringholz et al., 2014). The presence of the omega-3 fatty-acid-derived RvE1 in CF BALF correlates with better lung function compared to patients with undetectable RvE1 (Yang et al., 2012). Moreover, *P. aeruginosa* infection inhibits 15-epi-LXA4 production in HBE cells, thus promoting mucosal hyper-inflammation (Flitter et al., 2017; Recchiuti et al., 2019)

CO modulates several pathways in a way that may counteract the dysfunctions in the CF epithelium that lead to oxidative stress (Galli et al., 2012). These actions include inducing the aforementioned Nrf2-dependent and Nrf2-independent induction of HO-1, preventing ROS production downstream of NADPH oxidase activity, and rebalancing the defective glutathione homeostasis observed in CF cells (Gao et al., 1999; Velsor et al., 2001; Day et al., 2004; Galli et al., 2012; Yamamoto et al., 2014; De Bari et al., 2018).

## CO Stimulates Cellular Host Defense Against Infections: Implications in CF

Activating the HO-1/CO pathway or delivering exogenous CO has a promising additional clinical benefit. Namely, CO enhances MΦ bacterial killing capability and protects the lung epithelium from infection-associated damage (Chin and Otterbein, 2009). Several proposed mechanisms could explain how exposure to CO primes MΦs to better clear bacteria. For example, CO may stimulate bacterial uptake by redistribution of TLR4 at the plasma membrane (Otterbein et al., 2005). Moreover, the uptake of microorganisms such as *P. aeruginosa* by MΦs requires proper activation of the PI3K/AKT pathway (Araki et al., 2003; Bohdanowicz et al., 2010; Lovewell et al., 2014; Di Pietro et al., 2017; Demirdjian et al., 2018), which is stimulated by CO (Otterbein et al., 2000; Otterbein et al., 2003). TLR4 trafficking and its regulation at the plasma membrane of MΦs is altered in CF (Zhang et al., 2013). CF MΦs also show blunted induction of the PI3K/AKT signaling pathway in response to LPS or *P. aeruginosa* (Zhang et al., 2015; Di Pietro et al., 2017).

Wegiel et al. (2014a) have shown that modulation of the HO-1/CO pathway or exogenous delivery of CO increases the efficiency of MΦs in killing bacteria such as *Escherichia coli* and *Enterococcus faecalis*. The mechanism relies on CO promoting ATP production from bacteria, which, in turn, activates the Nacht, LRR, and PYD domains-containing protein 3 (NALP3) inflammasome system *via* binding of the purinergic receptor P2X7 (Wegiel et al., 2014a). An additional *in vivo* study has shown that CO-driven inflammasome activation facilitates bacterial clearance in a mouse model of polymicrobial infection caused by cecal ligation and puncture (Lancel et al., 2009). This may help macrophages kill *P. aeruginosa* clinical strains that are adapted to the altered CF environment (Riquelme et al., 2019). However, the effect of CO on inflammasome activation may depend on the type of stimuli, since Nakahira et al. have demonstrated that CO negatively regulates NLRP3 inflammasome activation in MΦs challenged with LPS but not with live bacteria (Jung et al., 2015).

Autophagy is another cellular mechanism that is fundamental to efficient bacterial clearance by immune cells. CO stimulates autophagy by inducing the expression and activation of the microtubule-associated protein 1A/1B-light chain 3 (LC3) (Lee et al., 2011). Autophagy is impaired in CF HBE (Luciani et al., 2010) and MΦs of CF patients (Abdulrahman et al., 2011). This contributes to the hyper-inflammation (Luciani et al., 2010) and poor bacterial killing of organisms such as *Burkholderia cepacia* (Abdulrahman et al., 2011) and *P. aeruginosa* (Ferrari et al., 2017). CO also increases acidification of the phagolysosome (Onyiah et al., 2013). Although controversial (Haggie and Verkman, 2007), this may be an additional dysregulated mechanism in CF MΦs that contributes to the failure to efficiently kill bacteria (Di et al., 2006).

Mitochondrial metabolic reprogramming is a key response by MΦs to efficiently fight pathogens during infection (Mills and O’Neill, 2016). CO affects mitochondrial function by binding to the cytochrome-c oxidase. At high doses, CO damages mitochondria. However, at physiological/non-toxic levels, CO has positive effects on mitochondrial metabolism. CO shifts the cellular energetic metabolism from glycolysis to oxidative phosphorylation and the pentose phosphate pathway, increasing oxygen consumption and ATP production. This may be a key mechanism in CO’s modulation of the MΦ response to infections. CO also induces mitochondrial and lysosomal biogenesis by activating the guanylate cyclase and the PI3K/AKT pathway, and upregulating transcription factors such as Nrf1, Transcription Factor A, mitochondrial (TFAM), Nrf2, Transcription Factor EB (TFEB), and PGC-1α (Zuckerbraun et al., 2007; Piantadosi et al., 2011; Queiroga et al., 2012; Wegiel et al., 2013; Motterlini and Foresti, 2017; Kim et al., 2018). These data suggest that cellular CO primes MΦs to better respond to cellular stressors. Although CO treatment has been associated with increased bacterial killing (Chin and Otterbein, 2009), caution is required when considering CO as a treatment for infection. This is because CO can inhibit the activity of NADPH oxidase 2 (NOX2) proteins. However, in other experimental settings, the beneficial effect of CO requires the presence of functional NOX2 (Nakahira et al., 2006; Lin et al., 2019). Thus, the inhibitory effect of CO on NADPH oxidases may depend on the CO dose used, cellular status (steady state or in response to stress), and type of stressor. This must be carefully evaluated when considering CO as a potential CF therapy because MΦs from CF patients may have an intrinsically lower capacity to activate NOX2 in response to bacterial infections (Assani et al., 2017).

A few *in vivo* studies suggest that administration of CO or CO-RMs protects against mortality after infectious challenge. Systemic delivery of CO-RMs reduced the mortality from 80% to 0% compared with controls in neutropenic mice infected with *P. aeruginosa*. This correlated with reduced bacterial recovery from the spleen due to either direct CO-mediated killing or enhanced bacterial clearance by the host immune system (Desmard et al., 2009). The authors demonstrate that mitochondrial function was protected in the CO-RM-treated mice and the pro-inflammatory response of sepsis was blunted (Lancel et al., 2009). Similarly, in a mouse peritonitis sepsis model, lower dose of CO-RM (10 mg/kg) resulted in an 80% survival rate. Additionally, upregulation of the HO-1/CO pathway, or delivery of inhaled CO, improved survival of mice in an *S. aureus* sepsis model. In this model, the mechanism of action relies on mitochondrial energetic metabolism reprogramming and biogenesis, increasing host cell survival, and countering the exuberant pro-inflammatory response in an AKT1-Nrf2 dependent manner (Macgarvey et al., 2012).

Finally, and particularly relevant in the context of CF lung disease, increased cellular levels of CO protect the bronchial epithelium from damage associated with *P. aeruginosa* infection. Secreted virulence factors from *P. aeruginosa* (e.g., *P. aeruginosa* quinolone signaling compound, PQS) decrease levels of HO-1 and Nrf2 expression in lung epithelial cells and primary HBE cells. This increases oxidative stress in epithelial cells, contributing to the pathogenicity of *P. aeruginosa* (Abdalla et al., 2017). In another study by Roussel et al., the *P. aeruginosa* biofilm-derived quorum sensing molecule N-(3-oxododecanoyl)-l-homoserine lactone (3OC12-HSL) decreased the activation of the Nrf2-HO-1 axis in HBE cells, increasing cellular ROS production (Roussel and Rousseau, 2017). Importantly, we and others have shown that stimulating the HO-1 pathway (with genetic manipulation (Zhou et al., 2004) or exposure to CO-RMs (Murray et al., 2012)) is sufficient to improve HBE cell survival following acute *P. aeruginosa* infection (Zhou et al., 2004) or exposure to *P. aeruginosa* biofilms (Murray et al., 2012). Improved CO cellular survival in response to infections may be mediated by stabilization of HIF-1α (Chin et al., 2007) and activation of the PI3K/AKT-driven induction of Bcl-2-mediated protection to apoptotic stimuli (Otterbein et al., 2003; Martin et al., 2004; Zhang et al., 2015; Shi et al., 2019).

## The Direct Effects of CO on Bacteria and Relevance to CF

Along with the previously discussed stimulation of the host cells, CO treatment has an additional potential clinical benefit in its direct bactericidal activity. Multiple studies have documented that CO and CO-RM treatment results in killing of a variety of pathogenic bacteria including *P. aeruginosa*, *E. coli*, *Salmonella enterica*, *S. aureus*, and *Helicobacter pylori* (Nobre et al., 2007; Murray et al., 2012; Bang et al., 2014; Rana et al., 2014; Flanagan et al., 2018). This has generally been true regardless of the carrier molecule used to deliver the CO, which accumulates inside bacterial cells before they release CO (Nobre et al., 2016). CO’s killing efficiency and mechanism of action against microorganisms likely differs based on the CO dose, microorganism, and the metabolic status of the microorganism (Chin and Otterbein, 2009; Chung et al., 2009; Tavares et al., 2012; Wilson et al., 2012). There are many explanations for how CO-RMs induce bacterial cell death (for a comprehensive review, see (Wilson et al., 2012)). One mechanism is that bacterial CO exposure generates reactive oxidative species (ROS) and subsequent DNA damage (Nobre et al., 2009). Another demonstrated mechanism is that CO directly targets respiration by binding to terminal cytochrome oxidases (Desmard et al., 2009; Tavares et al., 2013).

*P. aeruginosa* demonstrates the promises and limitations of CO and CO-RMs as antimicrobial therapies. We and others have shown that CO-RMs are effective at killing or aiding in the killing of *P. aeruginosa* clinical strains recovered from CF patients and grown in liquid culture or in biofilms (Murray et al., 2012; Flanagan et al., 2018). However, these studies also show that certain *P. aeruginosa* clinical isolates are less susceptible to CO (Murray et al., 2012; Flanagan et al., 2018). Moreover, the bacterial growth conditions influence CO’s effectiveness. CO does not effectively kill the common *P. aeruginosa* strain PAO1 in rich media, but it is highly effective in glucose-based media (Murray et al., 2012). CO-RMs also kill *P. aeruginosa* in anaerobic conditions (Desmard et al., 2009), similar to those found in the CF lung.

Riquelme & Prince recently pointed out that the metabolic environment is important for CF infections (Riquelme et al., 2019). The CF lung has high levels of succinate, and thus preferentially selects for *P. aeruginosa*, which efficiently metabolize succinate (Riquelme et al., 2017). This metabolic adaptation drives a transcriptional reprogramming of the bacteria, leading to expression of genes for extracellular molecules that favor bacterial biofilm formation. Interestingly, one study of a novel photo-activated CO-RM demonstrated CO-RM-dependent killing of *E. coli* when succinate was supplied as the sole carbon source (Nagel et al., 2014). Riquelme & Prince also provide evidence that *P. aeruginosa* clinical strains adapt to the altered CF environment and change the host immune response to induce recruitment of immune cells (monocytes and neutrophils) to the lungs, while retaining the ability to activate mitochondrial ROS. However, the recruited immune cells display immune dysfunction when challenged with CF-adapted *P. aeruginosa*, including failure to stabilize HIF-1α and to secrete IL-1β. Thus, CF-adapted bacterial isolates can evade clearance by MΦs (Riquelme et al., 2019). As discussed above (Wegiel et al., 2014a), by stabilizing HIF-1α and restoring IL-1β secretion, CO treatment may enhance the ability of host immune cells to better sense and eradicate *P. aeruginosa* isolates that are metabolically adapted to the CF lung environment.

## CO as a Modulator of Ion Channel Activity: Relevance to CF

CO is also emerging as a modulator of ion channel activity. CO regulates the function of the Ca^2+^-activated K (BK_Ca_), voltage-activated K+ (Kv), and Ca^2+^ channel (L-type) families, ligand-gated P2X receptors, tandem P domain K+ channels (TREK1), and the epithelial Na+ channel (ENaC). The mechanism/s by which CO modulates their activity is unclear. However, activation of the BK_Ca_ channel seems to be directly mediated by CO binding to a metal-based center in BK_Ca_ channels (Brazier et al., 2009; Telezhkin et al., 2011). Generation of cGMP by soluble guanylyl cyclase activation (Murad, 2006) and generation of mtROS (Wilkinson and Kemp, 2011; Peers et al., 2015) are two indirect mechanisms that have been proposed to mediate CO‐dependent modulation of ion channel activity.

### Relevance to CF

Loss of CFTR function leads to reduced expression of the inducible nitric oxide synthase, a target of CO, in CF murine and human airway epithelial cells (Kelley et al., 1997a; Kelley and Drumm, 1998; Elmer et al., 1999; Steagall et al., 2000). This reduces NO production, thus decreasing cGMP levels in CF cells and dysregulating transepithelial sodium and chloride transport (Elmer et al., 1999). Importantly, cGMPs activate CFTR in a PKA-independent manner (Kelley et al., 1997b), and promote trafficking of CFTR to the plasma membrane of the intestinal epithelium (Ahsan et al., 2017). Stimulating this pathway may also correct/potentiate mutant CFTR and thus ameliorate the intestinal fluid deficit in the CF intestine (Arora et al., 2017). Thus, by increasing cGMP cellular levels (Foresti and Motterlini, 1999), CO may help stimulate wild-type and mutant CFTR ion transport. A thought-provoking study by Wang suggests that CO may activate CFTR-dependent Cl^-^ and HCO_3_^-^ currents across the apical membrane of the rat distal colon. This study reported that the CFTR protein has a high-affinity ferric ion (Fe^3+^) binding site at the interface between the regulatory domain and intracellular loop 3. The binding of Fe^3+^ to CFTR prevents channel opening, and CO leads to release of the inhibitive Fe^3+^ ions, thus activating CFTR (Wang, 2017).

In addition to potentially regulating the CFTR function, CO also modulates the function of ion channels involved in CF lung disease, i.e., the large conductance calcium-activated potassium channels (BK_Ca_) and the epithelial Na+ channels (ENaC). The BK_Ca_ channels are expressed on the apical membrane of airways. Apical secretion of K^+^ provides a driving force for Cl^−^ flow, which maintains the airway surface liquid (ASL) volume. Its depletion leads to mucociliary dysfunction (Manzanares et al., 2011; Manzanares et al., 2015; Kim et al., 2020). Importantly, Salathe’s group recently reported that CF HBE cells have reduced BK_Ca_ channel activity due to increased lung inflammation, and that restoring BK_Ca_ channel activity reduces CF and inflammation-associated mucociliary dysfunction (Kim et al., 2020). Thus, CO’s ability to activate BK_Ca_ may reduce inflammation and help re-balance ion secretions in CF.

CO also inhibits the sodium channel ENaC, an established pharmacological target for CF lung disease (Mall, 2009). The ENaC channel absorbs Na^+^ from the apical side, thus reducing the ASL volume. CO inhibits ENaC in rat cultured alveolar type II cells and human airway epithelial cell line, and it prevents alveolar fluid reabsorption in perfused rabbit lungs (Althaus et al., 2009). Thus, CO can potentially block the hyperabsorption of sodium through the ENaC channel, which may restore ASL volumes (Mall et al., 2004). However, a different study has reported that CO has the opposite effect on ENaC in a mouse kidney cortical collecting duct cell line (Wang S. et al., 2009). These data are not easy to reconcile, but they may reflect tissue-specific differences in ENaC subunit composition, the CO doses, and experimental conditions. More studies are needed to clarify CO’s effect on ENaC in CF epithelium.

In summary, CO targets the signaling cascade associated with NO production and cGMP levels. It also directly targets the activity of ion channels such as BK_Ca_ (and potentially CFTR). It may thus help rebalance ion transport across the CF epithelium and restore physiological ASL levels.

## Exogenous Delivery of CO and Clinical Applications

Based on encouraging studies in preclinical models, the pharmacological use of CO has been tested in humans. In this rapidly evolving field, the current approaches to delivering controlled levels of CO in humans are: (a) inhalation and (b) a hemoglobin based-CO carrier.

### CO Inhalation

At high concentrations (10,000 ppm), inhalation of CO is toxic. However, at controlled low concentrations (10–200 ppm), exogenous CO delivery is safe in humans [NCT00531856 (Mayr et al., 2005) (Bathoorn et al., 2007)] and is beneficial against numerous diseases and pathological conditions featuring hyper-inflammation, tissue damage, pulmonary arterial hypertension, and ischemic conditions (Motterlini and Otterbein, 2010; Ji et al., 2016; Ryter and Choi, 2016; Ryter et al., 2018). Inhalation of 100–125 ppm CO by patients with stable chronic obstructive pulmonary disease (COPD) is safe, reduces sputum eosinophil levels and improves responsiveness to methacholine [NCT00122694 (Bathoorn et al., 2007)]. Results from a multicenter, double-blinded, clinical trial of inhaled CO in patients with idiopathic pulmonary fibrosis show that CO inhalation (100-200 ppm) was not associated with adverse events, but also did not result in significant changes in the study end points. These end points included differences in matrix metalloproteinase-7 serum concentration and pulmonary function test measures [NCT01214187 (Rosas et al., 2018)]. However, treatment with CO lead to a change in the expression profile of peripheral blood mononuclear cells dominated by oxidative phosphorylation-related genes (Casanova et al., 2019). Clinical implications for such transcriptional changes are not clear. Following successful preclinical studies in primates (Dalli et al., 2015; Fredenburgh et al., 2015), CO inhalation was tested in an initial safety study with patients with sepsis induced by acute respiratory distress syndrome (ARDS). Subjects were administered inhaled CO (100 ppm or 200 ppm) or placebo for 90 min for up to 5 consecutive days. The treatment was well-tolerated and appeared to be safe (Fredenburgh et al., 2018). A multi-center Phase II clinical trial of inhaled CO for the treatment of ARDS involving 5 US-based medical centers is currently ongoing (NCT03799874).

### Hemoglobin-Based CO Carrier

Prolong Pharmaceuticals has developed PP-007 (formerly known as *Sanguinate*), a polyethylene-glycol-modified (PEGylated) form of bovine hemoglobin loaded with CO. CO is released within 2 h of infusion and exchanged for oxygen, which is then delivered to areas of low oxygen tension (Abuchowski, 2017). This dual mode of action targets inflammation (CO) and hypoxia (O_2_), two complications in the CF lung. PP-007 is also PEGylated, which ensures stability and prolongs retention of the molecules in the circulation. PP-007 is being studied for several clinical situations to treat hypoxia and/or inflammation, including sickle cell disease (SCD), reperfusion injury, and cerebral hemorrhage (Misra et al., 2014; Abuchowski, 2016; Abuchowski, 2017; Dhar et al., 2017; Misra et al., 2017; Abu Jawdeh et al., 2018). PP-007 is safe in a phase I clinical trials in both healthy controls and patients with SCD. After a single intravenous dose (80, 120, or 160 mg/kg) in a randomized phase I single-blinded placebo-controlled study, the only observed adverse effect was a transient trend toward increased blood pressure, likely due to temporary intravascular volume expansion, which resolved within 24 h. In addition, a dose-dependent decrease in serum haptoglobin was observed, which binds to PP-007, forming a complex that is cleared from the circulation. Importantly, no Hb was detected in the urine, and no signs of nephrotoxicity were found (Abuchowski, 2016; Abuchowski, 2017). A second Phase I study in SCD patients also showed the transient increase in blood pressure, as well as an asymptomatic increase in troponin. Currently, PP-007 is being tested in several Phase II clinical trials in SCD (NCT02672540; NCT02600390). PP-007 is also well-tolerated in patients with other diseases, such as subarachnoid hemorrhage (SAH) (NCT02323685). PP-007 is FDA-approved for compassionate care, and has been successfully used as an artificial oxygen transfusion agent in two patients undergoing surgery, a patient with thrombotic thrombocytopenic purpura and a woman with postpartum hemorrhage (Holzner et al., 2018; Brotman et al., 2019). Patients were eventually stabilized, and no adverse events were reported (Sam et al., 2017; Thenuwara et al., 2017). Given PP-007’s cytoprotective effects and its use in ischemia-reperfusion injury in renal transplant patients (clinicaltrials.gov, ID: NCT02490202; Phase II/III), PP-007 may be able to prevent/reduce ischemia-reperfusion injury in CF patients after lung transplant (Porteous et al., 2015). The anti-inflammatory effects of PP-007 in preclinical mouse models of CF lung disease are currently being tested by our laboratory.

## Concluding Remarks

**Figure 5:**
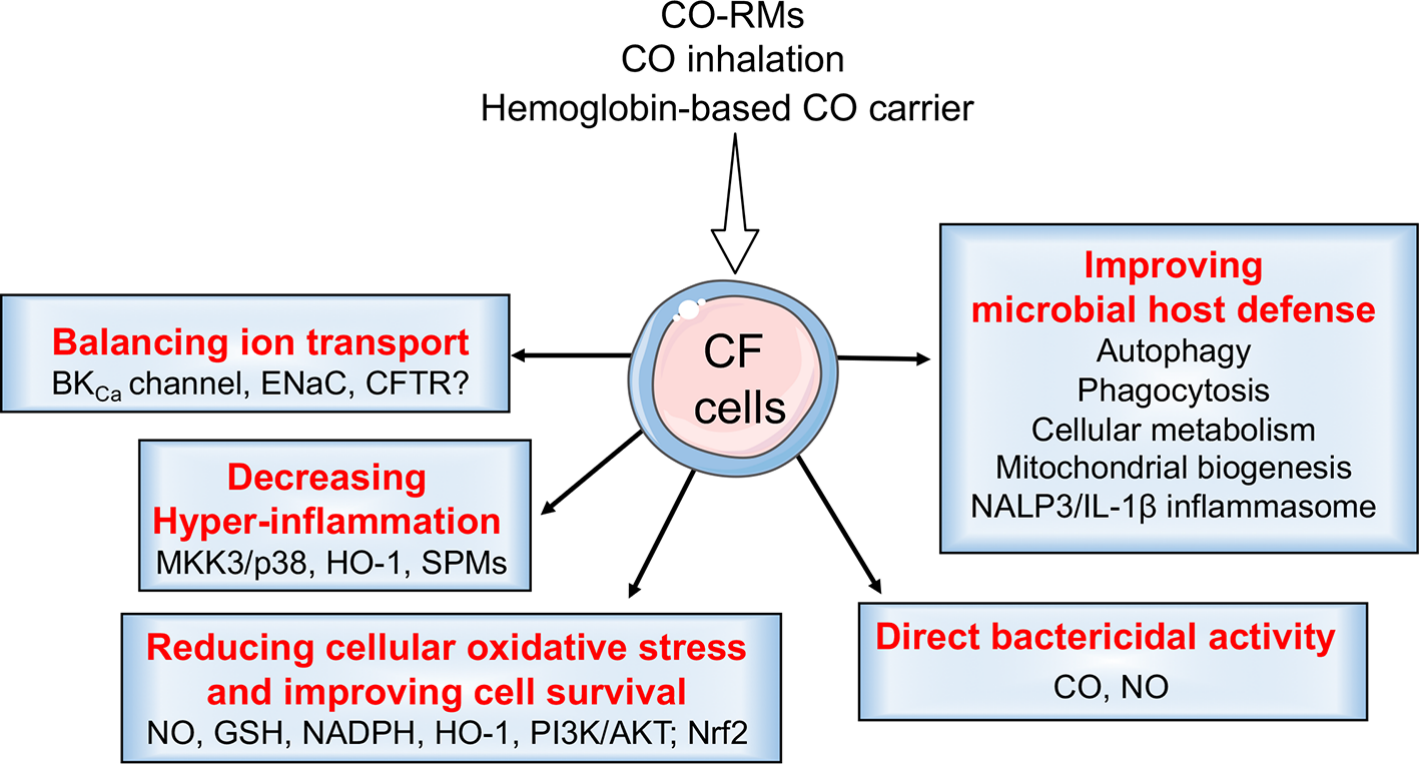
CO beneficial effects in cystic fibrosis (CF). CO may have several beneficial effects in CF. In addition to transcriptional induction of HO-1, CO helps to rebalance ion transport in bronchial epithelial cells by modulating the activity of BK_Ca_ channels, ENaC, and, possibly, CFTR. It has direct bactericidal activity and also primes the macrophages. It thus improves the host defense mechanisms by modulating autophagy, phagocytosis, the inflammasome, and immunometabolic responses. CO reduces cellular oxidative stress and improves cell survival by activating/inducing NO, GSH, NADPH, HO-1, PI3K/AKT, and Nrf2. CO also decreases hyper-inflammation by increasing levels of anti-inflammatory mediators, HO-1 and SPMs.

Modulating the HO-1/CO pathway, e.g., *via* CO administration, can attenuate hyper-inflammation, counteract oxidative stress, improve bacterial clearance by strengthening the host defense mechanisms or by direct killing, and rehydrate the airways (**Figure 5**). We therefore propose that the HO-1/CO pathway may be targeted as an adjuvant therapy to minimize lung disease in CF.

## Author Contributions

CP, HÖ, TM, and EB wrote the manuscript. CP designed the figures. EB initiated and overseen the work. All authors contributed to the article and approved the submitted version.

## Conflict of Interest

The Bruscia lab has been supported by a grant from Prolong Pharmaceuticals.

The authors declare that the research was conducted in the absence of any commercial or financial relationships that could be construed as a potential conflict of interest.
